# PAICS is related to glioma grade and can promote glioma growth and migration

**DOI:** 10.1111/jcmm.16647

**Published:** 2021-06-26

**Authors:** Baoshun Du, Zheying Zhang, Wenyu Di, Wenzhong Xu, Lei Yang, Shitao Zhang, Guoyang He, Rui Yang, Maode Wang

**Affiliations:** ^1^ Department of Neurosurgery The First Affiliated Hospital of Xi'an Jiaotong University Xi'an China; ^2^ Second Department of Neurosurgery Xinxiang Central Hospital Xinxiang China; ^3^ Department of Pathology Xinxiang Medical University Xinxiang China; ^4^ Department of Pathology The First Affiliated Hospital of Xinxiang Medical University Xinxiang China; ^5^ Department of Neurosurgery The Second Hospital Affiliated of Henan University of Science and Technology Luoyang China; ^6^ Department of Neurosurgery The Second Affiliated Hospital of Xi’an Medical University Xi'an China; ^7^ Department of Neurosurgery Xi'an No.3 Hospital The Affiliated Hospital of Northwest University Xi'an China; ^8^ Synthetic Biology Engineering Lab of Henan Province School of Life Science and Technology Xinxiang Medical University Xinxiang China

**Keywords:** glioma, grade, migration, PAICS, proliferation, weighted gene co‐expression network analysis

## Abstract

Glioma is a common malignant tumour of the brain. In this study, we aimed to investigate diagnostic biomarkers and its role in glioma. Weighted gene co‐expression network analysis (WGCNA) and Cytoscape software were used to screen the marker genes in glioma. RT‐qPCR and Western blotting methods were performed to determine the expression of PAICS, ERCC1 and XPA genes in glioma tissues. Expression level of PAICS in different grades of glioma was examined by immunohistochemistry. CCK8 and Colony formation assays were used to detect cell proliferation. Cell adhesion assay was used to detect adhesion ability. Wound healing and transwell tests were used to detect cell migration ability. Flow cytometry was used to detect cell cycle and apoptosis. According to the predicted co‐expression network, we identified the hub gene PAICS. Furthermore, we observed that PAICS expression level was up‐regulated in glioma tissues compared with normal tissues, and the expression level was correlated with the grade of glioma. Moreover, we found PAICS can promote glioma cells proliferation and migration in vitro. Flow cytometry results showed that si‐PAICS cells were stalled at the G1 phase compared with the si‐NC cells and knocking down PAICS expression can increase apoptotic rate. PAICS can regulate the mRNA and protein levels of nucleotide excision repair pathway core genes ERCC1 and XPA. l‐aspartic acid can affect the expression of PAICS and then inhibit glioma cell proliferation. Our results indicated that PAICS can promote glioma proliferation and migration. PAICS may act as a potential diagnostic marker and a therapeutic target for glioma.

## 1 INTRODUCTION

Malignant primary brain tumours are still poorly treated worldwide. Their 5‐year overall survival rate is <35%.^1^ Glioma is a very common primary brain tumour in adults, accounting for about 75% of all brain tumours.^2^ Gliomas are classified into four grades (I‐IV) according to the WHO brain tumour grading system.^3^ Grade IV has the highest degree of malignancy and is also known as glioblastoma. More than half of all gliomas are glioblastomas.^1^ Glioblastoma is the deadliest primary brain tumour in adults. Only 5% of patients survive more than 5 years. In recent years, despite significant advances in understanding the pathogenesis of glioma, effective treatments are still lacking. Therefore, in view of the high incidence, high mortality and lack of effective treatment of malignant glioma, it is necessary to find new therapeutic targets and effective interventions to improve patient survival.

To search for effective molecular targets in malignant gliomas, we used bioinformatics techniques to analyse the glioma data set GSE4412. The original authors mainly analysed significantly different genes with different survival rates. We wanted to identify the most effective hub genes by weighted gene co‐expression network analysis (WGCNA). WGCNA can cluster genes of the same expression type and analyse the relationship between modules and phenotypes.^4^ It is a method for analysing the expression patterns of multiple sample genes, that allows us to examine whether they are associated with diseases and other clinical features.^5^ It is widely used in research and is one of the most effective methods for co‐expression network analysis. The genes in the centre of the network constructed by WGCNA are known as hub genes. These genes are usually the key regulators and candidates that deserve our priority analysis and mining.^6^, ^7^

In this study, we elucidated that the hub genes phosphoribosylaminoimidazolesuccinocarboxamide synthase (PAICS) is over‐expressed in glioma tissues. Next, we found that PAICS can promote cell proliferation and migration, regulate the cell cycle and inhibit apoptosis. Moreover, we showed that PAICS can regulate key genes in the nucleotide excision repair (NER) pathway expression. In addition, we found that l‐aspartic acid can affect the expression of PAICS and thus inhibit the proliferation of glioma cells. Our results provide novel insights into the function of PAICS as a biomarker and a therapeutic target in glioma.

## 2 MATERIALS AND METHODS

### 2.1 Data processing

Data sets for WGCNA related to glioma were obtained from the National Center for Biotechnology Information (NCBI)’s Gene Expression Omnibus (GEO) database (http://www.ncbi.nlm.nih.gov/geo; accession number GSE4412). The data set contained a total of 85 samples; the microarray platform used is GPL96. According to the WHO criteria for glioma classification, 34 cases were of grade III and 51 cases were of grade IV. After downloading the data sets, we deleted the genes without annotation, after which there were 22 213 genes remaining. After merging the duplicate items, 13 515 remained. They were ranked according to their expression level, and the first 3000 genes were selected for analysis.

### 2.2 Construction of weighted gene co‐expression network

In order to make sure the reliability of the network establishment, the outlier samples were removed. There are six samples were removed (GSM99530 GSM99512 GSM99548, GSM99494, GSM99488 and GSM99542). And we chose the most suitable soft threshold (soft power = 4) according to the standard scale‐free network. Association of adjacent genes was calculated by a power function. Once the power value was determined, the module construction was continued with the WGCNA algorithm. WGCNA was run using the R package (http://www.r‐project.org/). Connection strength was plotted on heat maps using the R heatmap tool package.^8^, ^9^ The representation module was identified using the WGCNA algorithm. Module‐character relationships were estimated based on the association with the module eigengenes and clinical features. We found modules that were associated with glioma clinical traits by calculating the module eigengenes.^4^, ^10^ Modules with *P* < .05 were considered to be significantly correlated with clinical features.

### 2.3 Analysis of core genes in modules by Cytoscape

We selected the model genes that were highly correlated with the clinical features and imported them into Cytoscape to find the hub genes. Molecular complex detection, which finds clusters (highly interconnected regions) in a network,^11^ was used to screen for hub genes.

### 2.4 Tissue samples

A total of 10 pairs of glioblastoma samples and adjacent normal brain tissues were collected from Xinxiang Central Hospital, (Xinxiang, China) between June 2016 and December 2018, and the experimental scheme was examined and permitted by the Ethics Committee of Xinxiang Central Hospital. Experiments were performed consistent with guidelines and regulations of the Committee of Xinxiang Central Hospital. Informed consents were obtained from all patients. The collected tissue samples were frozen in liquid nitrogen and stored at −80°C. Tissues were examined, and the glioblastoma diagnosis was conﬁrmed. Classiﬁcations were based on the *WHO Classification of Central Nervous System Tumours* (revised fourth edition).

### 2.5 RNA extraction and RT‐qPCR

All the RNA was extracted with TRIzol reagent (Takara Bio Inc) refer to the manufacturer’s instructions. Reverse transcription into cDNA was performed with the Reverse Transcription Kit (Takara). RT‐qPCR was performed using SYBR Green I (Takara) in triplicate. Results were normalized to the expression of glyceraldehyde 3‐phosphate dehydrogenase (GAPDH). The primer sequences of PAICS were as follows: forward, 5′‐TTGCAGAAGAATAGCAACTGGTT‐3′ and reverse, 5′‐CACTGTGGGTCATTATTGGCAT‐3′. The primer sequences of excision repair cross‐complementation group 1 (ERCC1) were as follows: forward, 5′‐CAGGAGAGACGCCCAACCAG‐3′ and reverse, 5′‐CCAGCACATAGTCGGGAATTACG‐3′. The primer sequences of xeroderma pigmentosum group A (XPA) were as follows: forward, 5′‐CGGCTGCGGCTACTGGAG‐3′ and reverse, 5′‐AAGCCTCCTCCTGTGTCAATTATC‐3′. The primer sequences of GAPDH were as follows: forward, 5′‐GACTCATGACCACAGTCCATGC‐3′ and reverse, 5′‐AGAGGCAGGGATGATGTTCTG‐3′. The $2^{‐\Delta \Delta C_{t}}$ calculation method was used to calculate the relative expression levels of PAICS in tissues. ΔCt = ΔCtPAICS −ΔCtGAPDH, ΔΔCt = ΔCtTumour − ΔCtControl. The $2^{‐\Delta \Delta C_{t}}$ calculation method was also used for ERCC1 and XPA. Software of GraphPad Prism 6 was used to create graphs.^12^

### 2.6 Western blotting

Western blotting assay was performed as previously described. Antibody against PAICS (catalog number: A6450), ERCC1 (catalog number: A18066), XPA (catalog number: A1626) and GAPDH was purchased from ABClonal Inc. The protein expression level of PAICS, ERCC1 and XPA was normalized to GAPDH expression and quantified using Image J software.^13^

### 2.7 Tissue microarrays and immunohistochemistry

The tissue microarray was purchased from Xi'an Alena Biotechnology Ltd., Co. (catalog no. GL803c). Detailed clinicopathological data of the 80 patients are presented in Table S1. Remove one piece of tissue shedding and three medulloblastomas, and the rest is a total of 76 points. UltraSensitiveTMS‐P Ultra Sensitive Kit was purchased from Maixin Biotechnology Co., Ltd. (catalog number: KIT‐9720). Experimental procedures were performed according to the manufacturer’s protocols. The images were viewed using Aperio‐ImageScope 11.2.0708 software. The PAICS immunostaining score was calculated based on the percentage of positive stained tumour cells and staining intensity. The percentage positivity was ranged from 0 to 3, with 0 for <10%, 1 for 10%‐30%, 2 for 31%‐50% and 3 for >50%. The staining intensity was ranged from 0 to 3, with 0 for no staining, 1 for weakly stained, 2 for moderately stained and 3 for strongly stained. The positive percentage and staining intensity were scored in a double‐blind manner. The total score expressed by PAICS was calculated as percentage of positive score × staining intensity score, and the value range was 0 to 9.^14^

### 2.8 Establishment of PAICS knockdown cell lines and transfection

The siRNA interfering with PAICS expression was purchased from GenePharma. The siRNA‐1 nucleotide sequences of PAICS were 5′‐GCTGCTCAGATATTTGGGTTA‐3′. The siRNA‐2 nucleotide sequences of PAICS were 5′‐GUACACUGGUUGAUAUGAA‐3′. Cells were transfected with siRNA oligonucleotides using Lipofectamine 2000 (Invitrogen). The PAICS vector was purchased from Fenghui Biotechnologies Inc.

### 2.9 Cell Proliferation, wound healing and transwell assays

The proliferation and migration assays of transfected glioma cells were detected as previously described.^15^

### 2.10 Colony formation

When the cells grow well, they are trypsinized. Inoculate 200‐300 cells in a six‐well plate and place them in an incubator at 37°C and 5% CO2 for about 2 weeks. When cell clones are visible to the naked eye, paraformaldehyde fixes the cells for 15 minutes, and then Giemsa staining for about 20 minutes. After drying and the number of colonies formed was counted with naked eyes (≥50 cell clones).

### 2.11 Cell adhesion assay

5000 cells per well were seeded into a 96‐well plate precoated with matrigel (BD, Cat. #356234). After 2 hours of culture, the cells were gently washed with PBS to remove the non‐adherent cells. The attached cells were fixed under a 200 magnification microscope and photographed for counting.^16^

### 2.12 Flow cytometry

24 hours after transfection, the cells were collected. Fluorescein isothiocyanate (FITC) Annexin V and propidium iodide were used to stain cells in line with the FITC Annexin V Apoptosis Detection Kit (BD Biosciences) protocol. Then, ﬂow cytometric analysis was used to detect numbers of cell in G0‐G1, S, G2–M phases and apoptosis.^17^

### 2.13 Statistical analysis

Statistical analysis was performed using SPSS 20.0 software. Data are showed as the mean ± standard deviation of at least three independent experiments. Differences between the two groups were evaluated by two‐tailed Student’s *t* test. *P* < .05 was considered as statistically significant difference.

## 3 RESULTS

### 3.1 Data sets processing of the glioma

The 3000 genes were further for hierarchical clustering analysis, which was carried out with the function ‘flashClust’. We identified that the final data set could be divided into three groups in our cluster analysis (Figure S1). Before constructing the co‐expression network, we calculated an appropriate weighted parameter of adjacent function, namely soft threshold. We selected the correlation coefficient close to 0.9, soft threshold = 4, to construct gene modules with the WGCNA package (Figure S2).

### 3.2 Association of each module and clinical features

**FIGURE 1:**
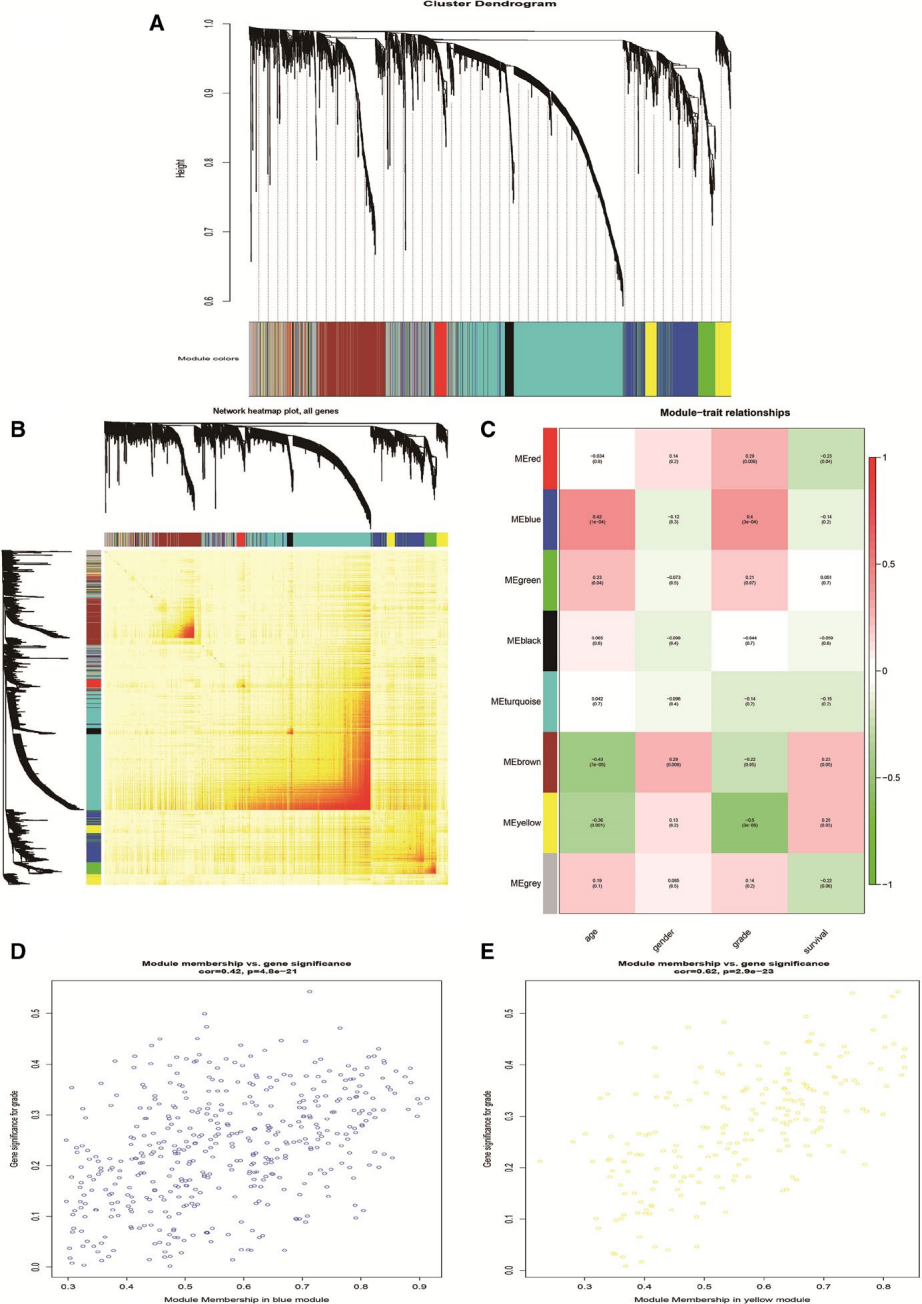
The correlation between genes and clinical characteristics was analysed. A, Clustering with dynamic tree cutting method, we found gene modules with different characteristics. B, Heatmap plot of topological overlap in the gene network. In the heat map, row and column correspond to gene modules, light colour denotes low topological overlap, and deeper colour denotes higher topological overlap. C, Correlation analysis between characteristic genes and clinical characteristics. Each row corresponds to the module feature geneswerelisted as a clinical trait. Each cell contains correlation and *P* value. D, Correlation analysis between characteristic genes and clinical characteristics of blue module. E, Correlation analysis between characteristic genes and clinical characteristics of yellow module

Eight modules were constructed and depicted in different colours based on WGCNA, and genes that did not belong to any modules were placed in the grey module. The grey gene module was neglected in this study (Figure 1A). We calculated the correlation matrix and adjacency matrix of the gene expression profile, converted them into a topological overlap matrix and obtained the system cluster tree of genes. Results indicated that the blue module overlapped with a deeper colour (Figure 1B). Specific expression modules were associated with clinical features and the correlation between the modules and the four clinical traits of age, sex, grade and survival were calculated to detect which clinical trait the module was related to. The results indicated that there were two modules, blue and yellow were significantly associated with the grade of glioma. Among the correlations of all clinical traits and gene modules, the positive correlation was strongest for blue module and grade. (Figure 1C). In addition, we plotted scatter graphs of the gene significance for grade and module members, and the results were same as earlier (Figure 1D,E). We selected the blue module genes, which were positively correlated with clinical features, for further analysis.

### 3.3 Visualization of hub genes in the module

We imported genes contained in the blue module and correlation file to Cytoscape software to analyse the core genes. We found the top three core genes that contained PAICS, EGR1 and HLA‐F. The core genes in the three modules are represented by yellow (Figure S3).

### 3.4 Expression of PAICS is up‐regulated in glioblastoma tissues

**FIGURE 2:**
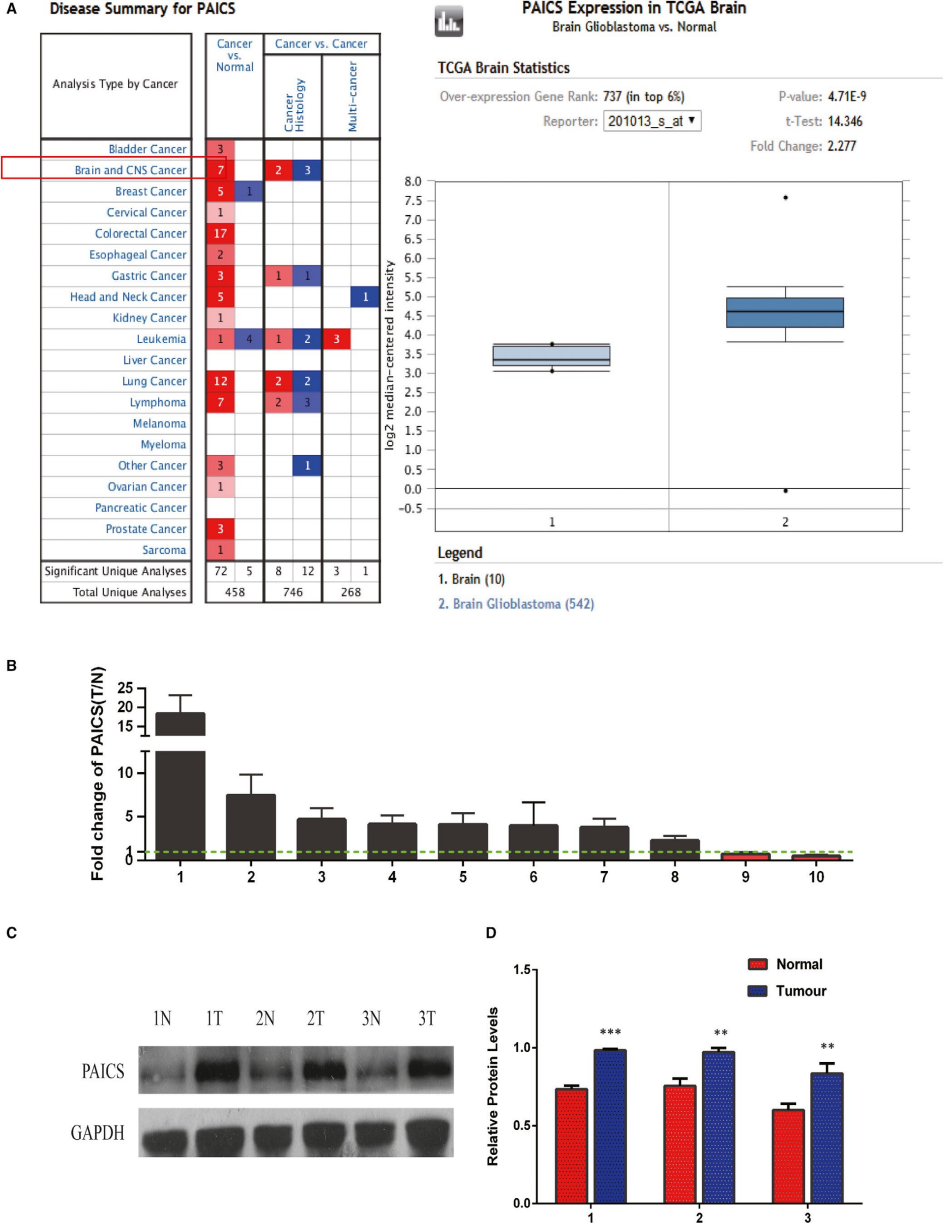
Expression levels of PAICS in glioblastoma tissues. A, Expression levels of PAICS mRNA with glioma tissues and control tissues in Oncomine. Red boxes represent expression down‐regulation. The graph on the right is the results from TCGA (http://cancergenome.nih.gov/) database. TCGA is a powerful database platform for integrating multiple cancer genome sequencing data. The results showed that PAICS expression was up‐regulated 2.277‐fold in glioma specimens compared with control brain tissue specimens. B, RT‐qPCR analysis of PAICS expression in glioblastoma tissues compared with the controlbrain tissues. Error bars indicate the mean ± standard deviation of three independent experiments. (N: normal brain tissue, T: glioma tissue, red column represents T/N less than 1). C, Western blot analysis of PAICS protein expression in three pairglioblastomatissues compared with the normal tissues. The differences between tumour group and normal groupwere tested by using independent‐samples *t* test. D, Protein expression level of PAICS was normalized to GAPDH and quantified using Image J. Error bars represent the mean ± SD of three different tests. **P < .01;***P < .001

PAICS expression level was significantly up‐regulated in glioma tissues compared with that in adjacent normal tissues, based on Oncomine microarray data set and web data‐mining online platform results (Figure 2A). We further detected the mRNA expression levels of PAICS in 10 pairs of glioblastoma tissues and normal brain tissues. Compared with the normal tissues, the expression level of PAICS was up‐regulated in glioblastoma tissues (Figure 2B). Western blotting was used to detect the protein level of PAICS in three paired glioblastoma tissues. Results displayed that the protein expression of PAICS in glioblastoma tissues was higher than that in paired normal tissues (*P* < .05) (Figure 2C,D).

### 3.5 Expression of PAICS protein in glioma of different grade

**FIGURE 3:**
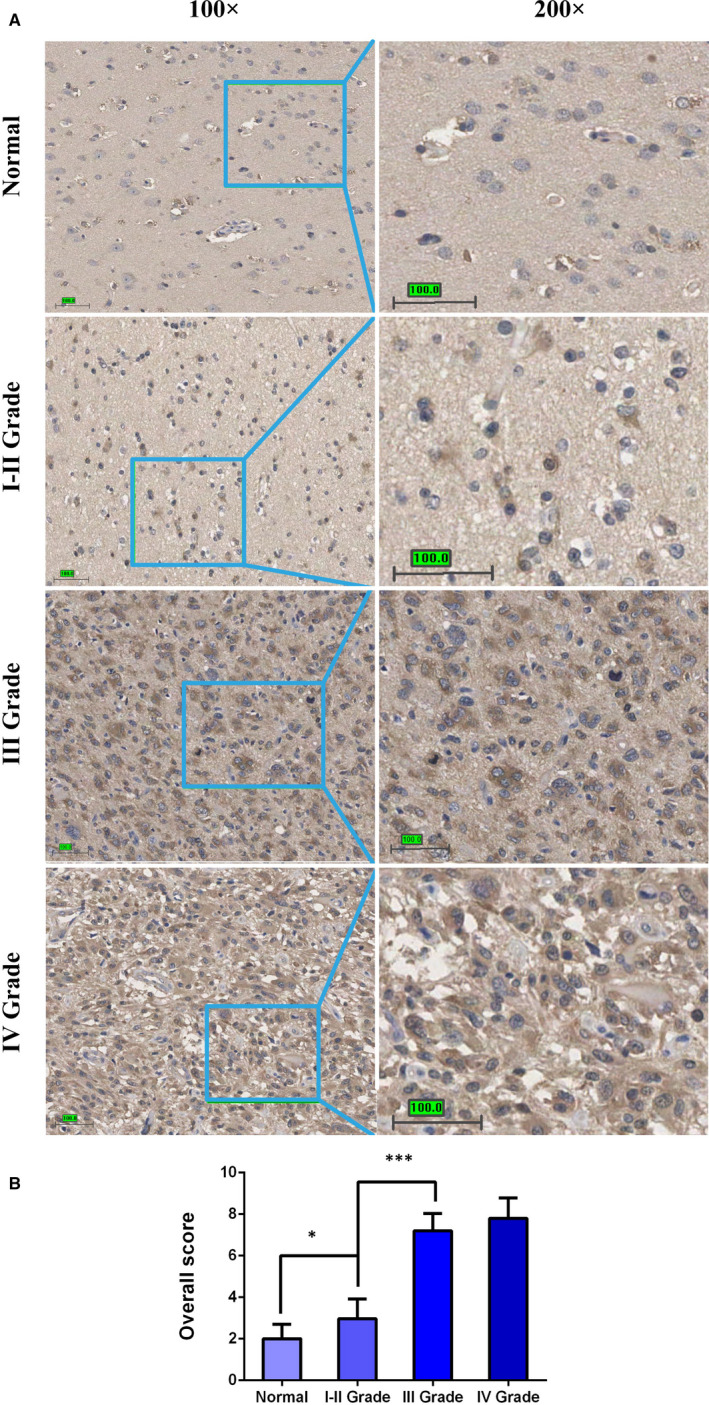
Expression levels of PAICS protein in different glioma tissues. A, Representative PAICS staining in I‐II grade, III grade, IV grade glioma tissues and adjacent normal brain tissues. Scale bar, 100 µm. B, The PAICS score was calculated as introduced in materials and methods part. *P < .05;***P < .001

To investigate the role of PAICS in different grade glomia, the expression levels of PAICS protein were detected in five normal brain samples, 31 I‐II grade samples, 41 III and IV grade glioma samples from 77 patients using IHC analysis. We observed that PAICS protein expression was increased in I‐II grade glioma tissues compared with in the normal tissues (*P* < .05) and significantly higher in grade III‐IV tissues than in normal tissues (*P* < .001). Furthermore, there was a significant difference between grade III and I‐II (*P* < .001), but no significant difference between grade III and IV (Figure 3A,B).

### 3.6 Inhibition of PAICS suppresses proliferation and migration in glioma cell lines

**FIGURE 4:**
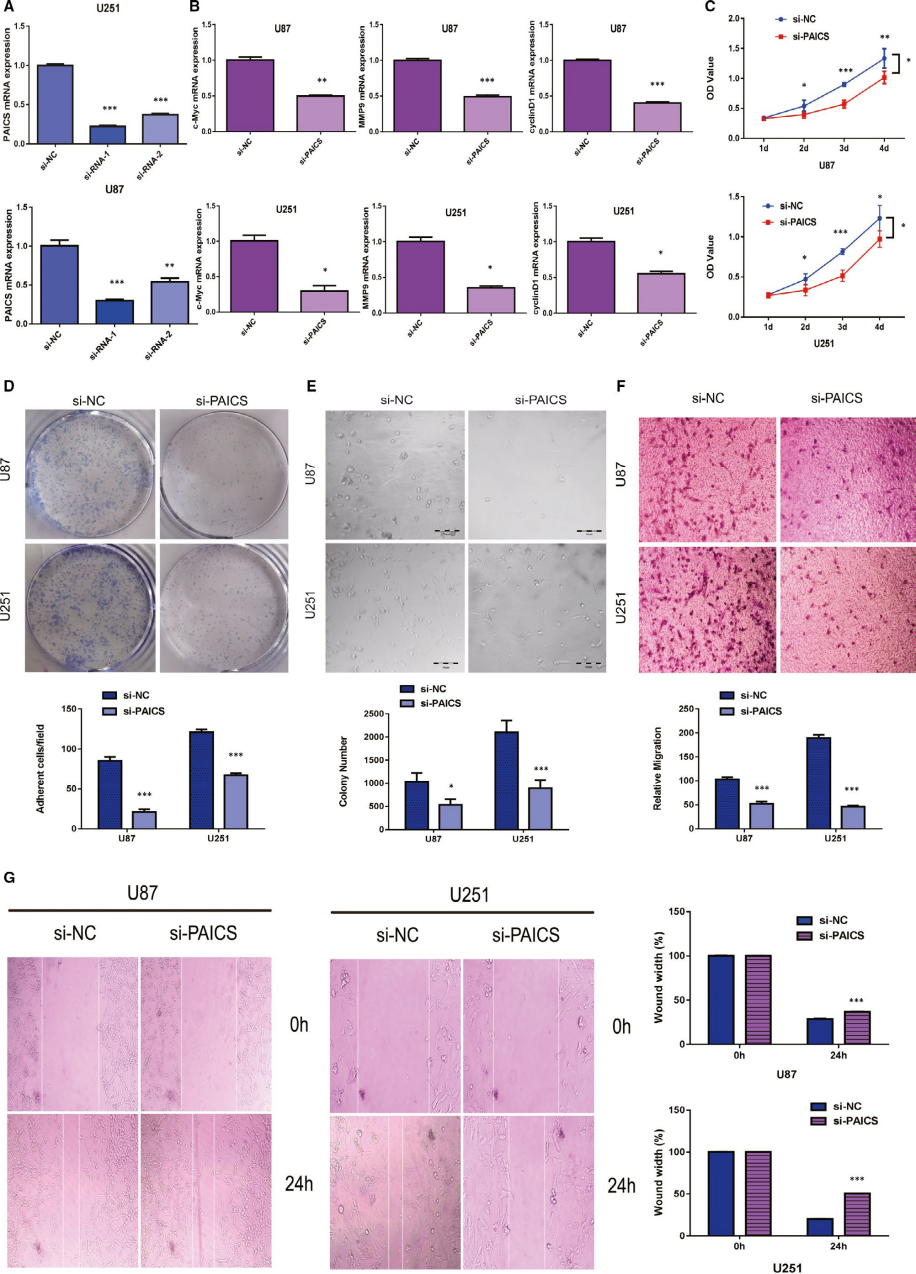
Inhibition of PAICS in glioma cells weakened the cell growth and metastasis in vitro. A, RT‐qPCR analysis of PAICSmRNA levels after the transfection of the si‐NC or si‐PAICSsmall interfering RNAs. PAICS expression was normalized to GAPDH, Error bars indicate mean ± SD of three independent experiments. **P < .01; ***P < .001. B, RT‐qPCR analysis of c‐Myc, MMP9 and cyclin D1 mRNA expression levels after the transfection of the si‐NC or si‐PAICSsmall interfering RNAs. PAICS expression was normalized to GAPDH. Error bars indicate mean ± SD of three independent experiments. *P < .05;**P < .01; ***P <.001. C, Knockdown of PAICS inhibited cell proliferation on the basis of CCK8 assays. Error bars represent the mean ± SD of five independent experiments. *P < .05; **P < .01;***P < .001. D, Knockdown of PAICS inhibited colony formation. Error bars represent the mean ± SD of three independent experiments. *P < .05; ***P <.001. E, Knockdown of PAICS inhibited cell adhesion. Error bars represent the mean ± SD of three independent experiments. ***P < .001. F, Inhibition of PAICS decreased cell migration as determined by transwell assays. The bar chart represents the migration cell numbers. Error bars represent the mean ± SD of five different field. ***P < .001. G, Inhibition of PAICS decreased cell migration as determined by wound healing assay. The bar chart represents the percentage of distance at 24 h divided by the distance at 0 h. Date are presented as mean ± SD of three independent experiments. ***P < .001

To understand PAICS functionality in glioma progression, we knocked down the PAICS expression with siRNA‐309 and siRNA‐424 in U87 and U251 cells, respectively, and screened out the siRNA‐424 with the highest efficiency (Figure 4A). qRT‐PCR assay was used to determine the expressions of c‐Myc, MMP9 and cyclinD1 genes related to proliferation and migration. The results showed that the expression of genes related to proliferation and migration was reduced after PAICS knockdown (Figure 4B). CCK8 and Colony formation results showed that knockdown of PAICS expression level reduced cell growth (Figure 4C,D). Furthermore, cell adhesion showed that reduce PAICS expression level weakened cell adhesion (Figure 4E). The enhancement of the ability of clone formation and adhesion may promote cell migration. Transwell and wound healing assay were used to detect ability of migration, the results showed that inhibition of PAICS could reduce the migration ability of U87 and U251 cells (Figure 4F,G).

### 3.7 Inhibition of PAICS suppresses cell cycle and apoptosis in glioma cell lines

**FIGURE 5:**
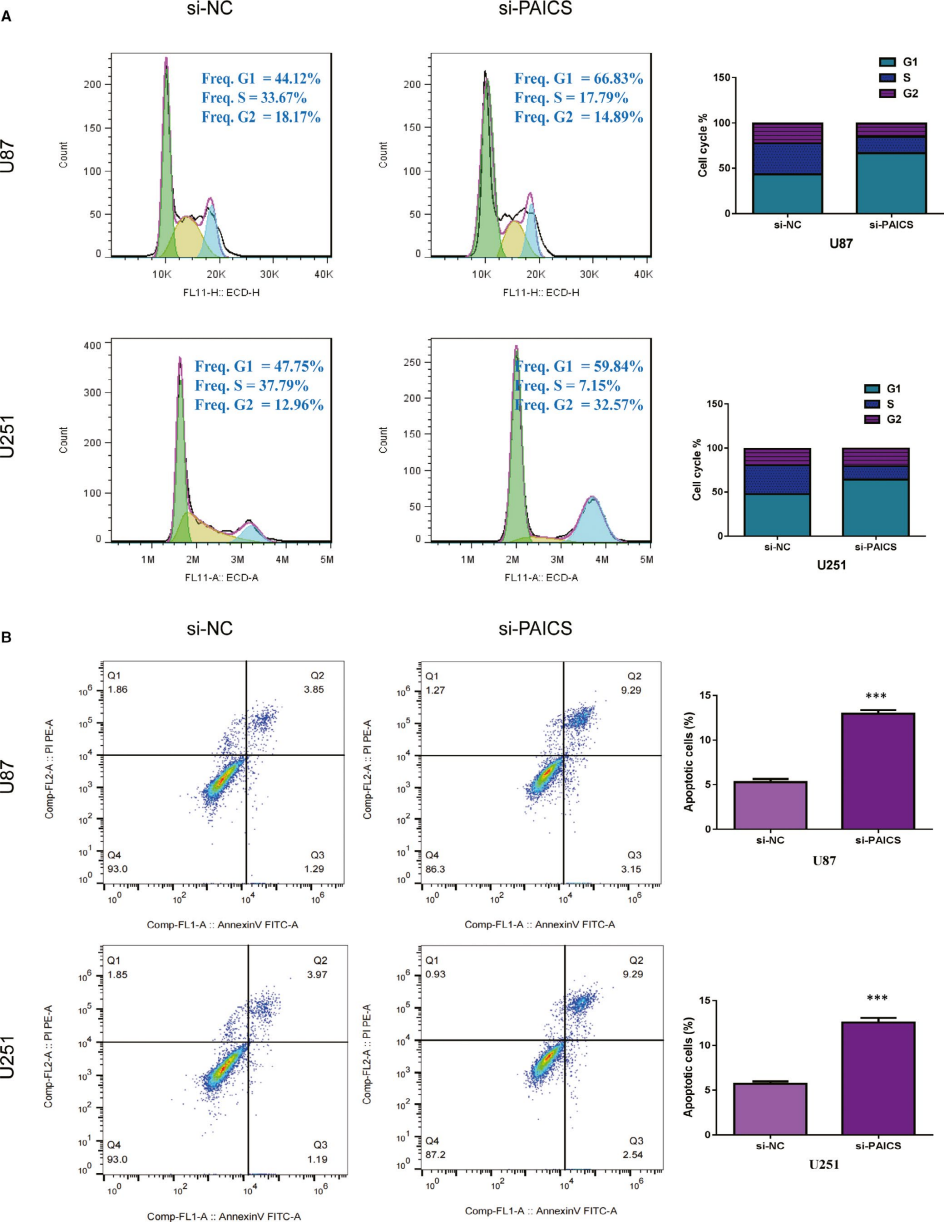
Knockdown of PAICS in glioma cells inhibit cell cycle and promote apoptosis in vitro. A, Cell cycle was analysed by flow cytometry after knocking down PAICS. The results showed that PAICS depletion led G1 arrest. B, Cell apoptosis was analysed by flow cytometry after knocking down PAICS. The results showed that PAICS depletion leading to an increased rate of apoptosis. ***P < .001

Flow cytometry revealed that decrease the expression of PAICS in U87 and U251 cells promoted the proportion of cells in G0/G1 phase, while inhibited the proportion of glioma cells in S phase (Figure 5A). Furthermore, we found that decrease the expression of PAICS increasing apoptotic rate of glioma cells (Figure 5B). Decreasing cell cycle division and increasing cell apoptosis may be one of the reasons that PAICS promotes tumour proliferation.

### 3.8 PAICS regulates the genes expression through NER pathway

**FIGURE 6:**
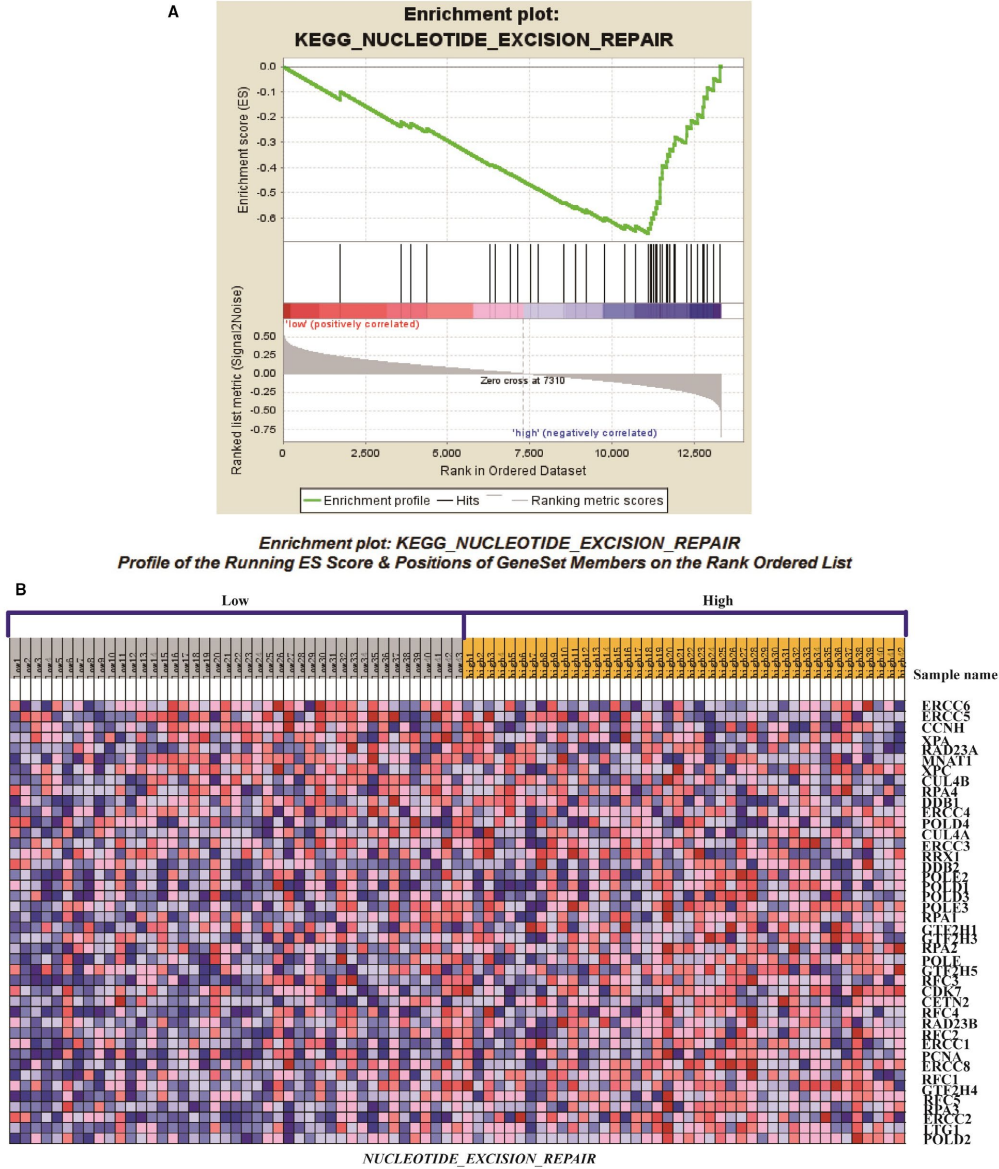
Gene set enrichment analysis (GSEA) for PAICS. A, Microarray data for glioma samples GSE4412 (n = 85) were analysed using GSEA software to identify significant gene sets. The NER signalling related genes was enriched (*P* < .05). B, GSEA‐generated heat map for highly enriched genes in PAICS high‐expression samples vs PAICS low‐expression samples

**FIGURE 7:**
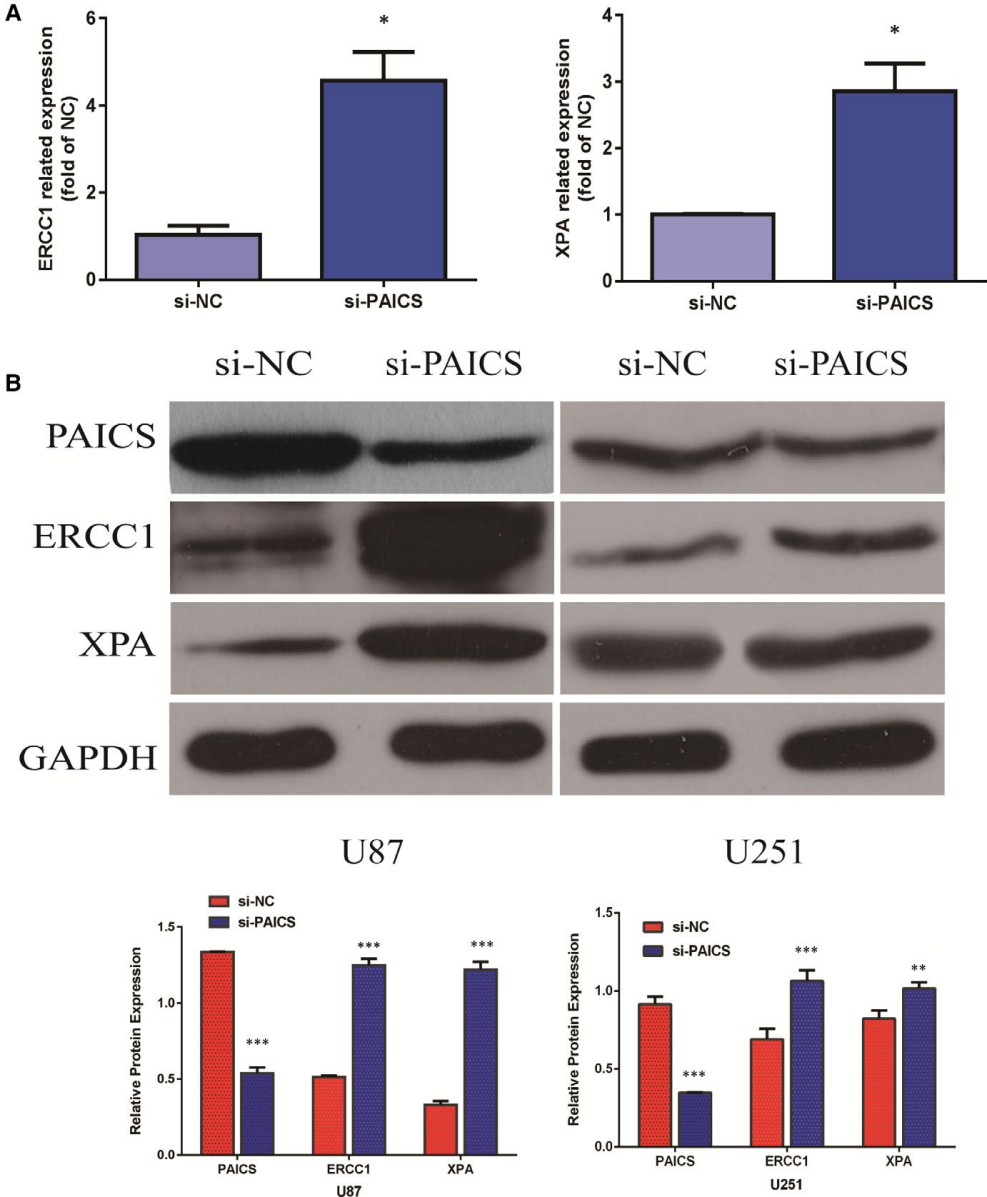
PAICS regulates the genes expression of NER pathway. A, The expression of ERCC1 and XPA in U87 and U251 cells was detected by qRT‐PCR after inhibiting the expression of PAICS. ERCC1 and XPA expression level was normalized to GAPDH. Error bars represent the mean ± SD of three independent experiments. The differences between independent experimental groups were tested by using independent‐samples *t* test. *P < .05. B, The expression of PAICS, ERCC1 and XPA in U87 and U251 cells was detected by Western blot after decreasing the expression of PAICS. GAPDH served as the loading control. Error bars represent the mean ± SD of three independent experiments. The differences between independent experimental groups were tested by using independent‐samples *t* test. **P < .01; ***P < .001

To examine how PAICS is playing a role in glioma progression, we performed a GSEA gene enrichment analysis on the GSE4412 samples and found that NER signal pathway enrichment was most significant when PAICS was highly expressed (Figure 6A,B). Then, we determine the mRNA and protein expression levels of ERCC1 and XPA which is the key genes in NER pathway. The result showed that the mRNA level of ERCC1 and XPA was increased after inhibiting the expression of PAICS (Figure 7A). Similarly, the protein level of these genes was also increased after knocking down the expression of PAICS (Figure 7B). We hypothesized that PAICS could modulate NER pathway signalling. There is a negative correlation between them.

### 3.9 l‐aspartic acid inhibits proliferation of glioma cells by regulating the expression of PAICS

**FIGURE 8:**
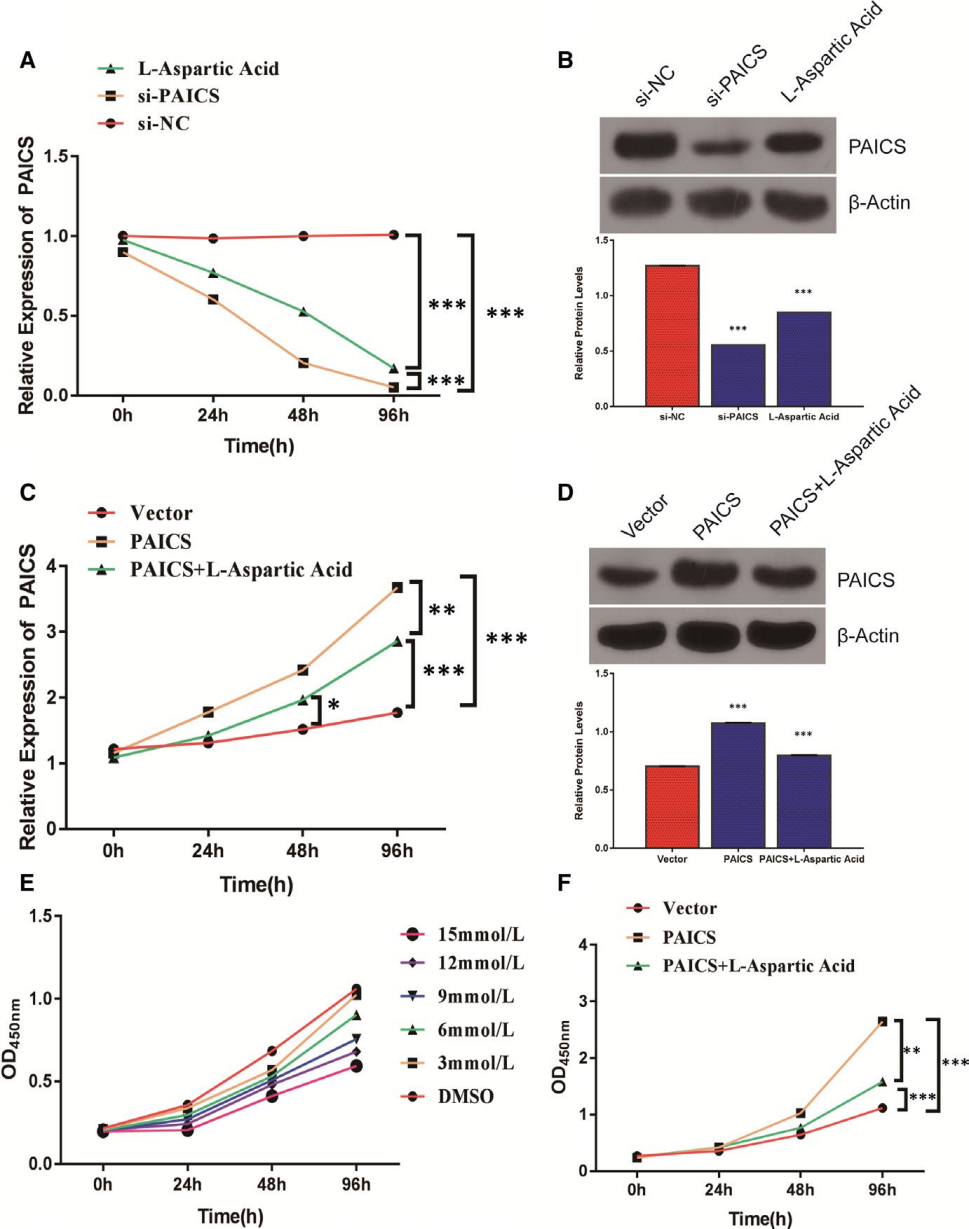
l‐aspartic acid inhibits proliferation of glioma cells by regulating the expression of PAICS. A, At 0, 24, 48 and 96 h, RT‐qPCR was used to detect PAICS gene expression in the l‐aspartic acid group, the PAICS interference fragment group and the control group were detected, respectively. ***P < .001. B, At 0, 24, 48, and 96 h, Western blot was used to detect PAICS gene expression in the diffient group. ***P < .001. C, RT‐qPCR was performed to detect PAICS expression after adding l‐aspartic acid. *P< .05; **P < .01; ***P < .001. D, Western blot was performed to detect PAICS expression after adding l‐aspartic acid. ***P < .001. E, Effects of different concentrations of l‐aspartic acid (3, 6, 9, 12 and 15 mmol/L) and DMSO on cell proliferation were determined by CCK8 cell proliferation assay. F, CCK8 was performed to detect PAICS proliferation in different group. **P < .01; ***P < .001

The drug l‐aspartic acid for PAICS targets was queried through the Drugbank database. First, we verified the effect of l‐aspartic acid on PAICS expression, the results showed that both l‐aspartic acid and PAICS interfering fragments can reduce the expression of PAICS, but l‐aspartic acid is not as efficient as interfering fragments (*P* < .001) (Figure 8A,B). We transfected glioma cells with an overexpressing PAICS vector (Figure S4). l‐aspartic acid can reverse the high expression of PAICS induced by PAICS vectors (*P* < .01) (Figure 8C,D). Then, we examined the effect of different concentrations of l‐aspartic acid (3, 6, 9, 12 and 15 mmol/L) on the proliferation of glioma cells. We found significant differences among each groups (*P* < .05) (Figure 8E). We further tested the effect of l‐aspartic acid on PAICS‐induced cell proliferation and found that l‐aspartic acid could inhibit the proliferation of glioma cells induced by PAICS (Figure 8F).

## 4 DISCUSSION

Glioma is a glial or precursor cell originating from the neuroectoderm, including astrocytoma, oligodendroglioma and ependymoma. According to the WHO brain tumour grading system, glioma are classified into four grades: I, II, III and IV. Due to the complex pathogenesis of glioma, there is no effective strategy for the treatment of glioma at present.

Weighted gene co‐expression network analysis method can be used to cluster genes with highly similar expression profiles, classify them into different modules and identify modules associated with clinical features. In order to find candidate gene clusters in glioma, we collected four clinical characteristics of age, sex, grade and survival and constructed a gene co‐expression network. Due to the limitation on the number of input genes, we selected the first 3000 genes that have the largest differences. WGCNA method was applied to analyse the expression of 3000 different genes (in 85 samples) obtained from the NCBI. After analysis of these genes, we found that the *P* values of three modules associated with tumour grade were <0.05. The modules were blue, red and yellow. Blue and red modules were positively correlated with tumour grade, while yellow module was negatively correlated with this clinical trait. Among the correlations of all clinical traits and gene modules, the positive correlation was strongest for blue module and grade. We selected the blue module gene with a smaller *P* value for further analysis. The protein interaction network of genes was constructed by introducing the genes into the STRING database, and the gene phosphoribosylaminoimidazole carboxylase and PAICS with the highest score was identified by Cytoscape software analysis.

The PAICS gene encodes a bifunctional enzyme whose N‐terminal region exhibits phosphoribosaminoamidazole carboxylase activity and C‐terminal region contains PAICS, which catalyses the biosynthesis of purines.^18^ In addition to its catalytic activity, PAICS also has a protein‐binding function.^19^ Previous studies have revealed that PAICS plays an important role in various tumours and is associated with tumour proliferation and metastasis.^20^, ^21^, ^22^ However, the expression and role of PAICS in glioma have never been reported. A data set of a large number of glioma samples from the Oncomine database showed that the expression of PAICS in glioma tissues was obviously up‐regulated compared with that in normal tissues. To confirm the results of bioinformatics analysis, we detected PAICS expression in glioblastoma and normal tissue samples by RT‐qPCR and Western blotting. The results were consistent with bioinformatics analysis. Then, we detected the expression of PAICS in gliomas of different grades and found that the expression of PAICS in gliomas of grade III and IV was significantly increased compared with normal brain tissue and gliomas of grades I‐II. There were also differences between grades I‐II and normal brain tissue, but there were no significant differences between grades III and IV. These results suggest that PAICS expression is associated with glioma grade. Furthermore, we detected the expression of c‐Myc, MMP9 and clyclin D1 genes related to proliferation and migration by PCR, and found that the expression of c‐myc, MMP9 and clyclin D1 was related to proliferation and migration. Through CCK8, plate cloning, cell adhesion, transwell and wound healing test, we further confirmed that PAICS can promote the proliferation and migration in glioma cells. PAICS may promote the migration of tumour cells by increasing the number of clones and enhancing the adhesion ability. Flow cytometry results showed that interfering with PAICS could promote cells to stay in G1 phase and reduce the proliferation of tumour cells. Interference with PAICS can also increase the apoptotic rate of cells. Decreasing cell cycle division and inhibiting cell apoptosis may be one of the reasons that PAICS promotes tumour proliferation.

We further explore the mechanism of PAICS. GSEA gene enrichment analysis of samples shows that NER signalling pathway enrichment is most significant when PAICS is highly expressed. We tested key genes ERCC1 and XPA in the NER pathway. It was found that when PAICS expression was inhibited, and mRNA and protein of these key genes were all up‐regulated. There was a negative correlation between the NER pathway and PAICS expression. Some papers have reported that the low expression of NER pathway‐related genes increases the incidence of tumours.^23^, ^24^ NER is an important way to repair DNA damage. DNA damage caused by ultraviolet rays and chemotherapy drugs is mainly repaired by NER pathway. ERCC1 is considered a leader gene of the NER system and is responsible for identifying and cutting damage, while XPA continue the repair process immediately after the ERCC1. The NER pathway gene expression is reduced in tumours, and the gene repair ability is low, thus increasing the risk of tumours. Our results showed that PAICS is a cancer‐promoting gene that is up‐regulated in tumours and PAICS may promote cancer cells proliferation and migration through inhibiting NER pathway. When PAICS is inhibited, the NER pathway gene is up‐regulated and DNA repair capacity is enhanced, which may inhibit further tumour development.

We found the drug l‐aspartic acid for the PAICS target through the Drugbank database. l‐aspartic acid can inhibit liver cancer cell proliferation by inhibiting AKT phosphorylation, and no related reports were found in glioma.^25^ We speculate that l‐aspartic acid also inhibits cell proliferation in gliomas. The experimental results also support our hypothesis that as the concentration of l‐aspartic acid increases, the effect of inhibiting the proliferation of glioma cells becomes more and more obvious, which is significantly different from the control group. Through the above results, we speculated that PAICS might play an important role in glioma. The mechanism of PAICS in glioma is not yet clear and requires further investigation.

## CONFLICT OF INTEREST

The authors declare that they have no conflicts of interest with the contents of this article.

## AUTHOR CONTRIBUTIONS

**Baoshun Du:** Conceptualization (equal); Data curation (equal); Methodology (equal); Writing‐original draft (equal). **Zheying Zhang:** Conceptualization (equal); Data curation (equal); Methodology (equal); Software (equal); Validation (equal); Writing‐original draft (equal). **Wenyu Di:** Data curation (supporting); Formal analysis (supporting); Investigation (supporting); Methodology (supporting); Writing‐original draft (supporting). **Wenzhong Xu:** Data curation (supporting); Formal analysis (supporting); Methodology (supporting). **Lei Yang:** Formal analysis (supporting); Investigation (supporting); Methodology (supporting); Resources (supporting). **Shitao Zhang:** Conceptualization (supporting); Formal analysis (supporting); Investigation (supporting); Writing‐review & editing (equal). **Guoyang He:** Data curation (supporting); Formal analysis (supporting); Methodology (supporting). **Rui Yang:** Data curation (supporting); Investigation (supporting); Methodology (supporting); Writing‐original draft (supporting). **Maode Wang:** Formal analysis (equal); Visualization (equal); Writing‐original draft (lead); Writing‐review & editing (lead).

## Supporting information

Fig S1

Fig S2

Fig S3

Fig S4

Table S1

## Data Availability

The data that support the findings of this study are available from the corresponding author upon reasonable request.
