# Supplementary figures and images for: PAICS is related to glioma grade and can promote glioma growth and migration
Source: J Cell Mol Med. 2021 Jun 26;25(16):7720–33. doi: 10.1111/jcmm.16647 (PMC8358864; doi:10.1111/jcmm.16647)

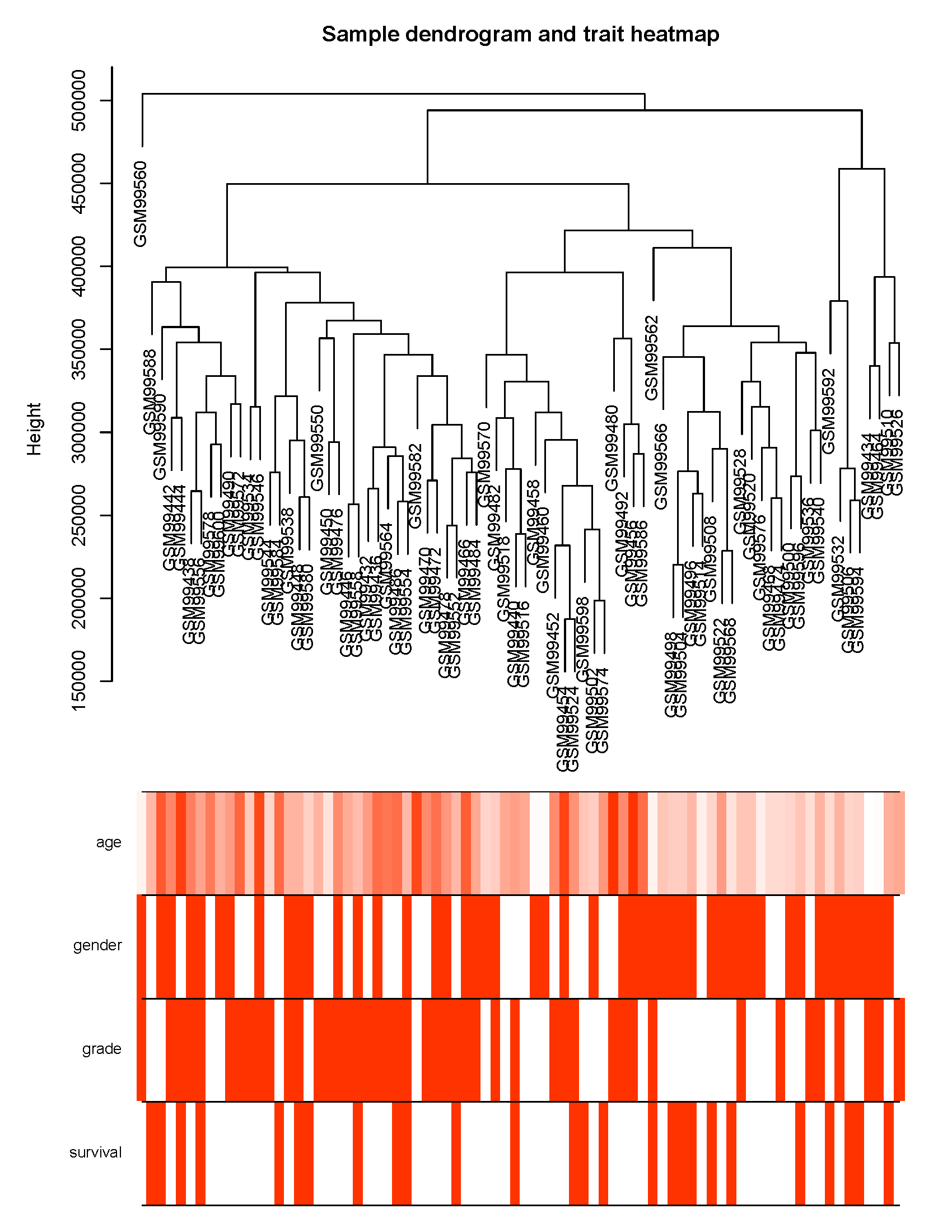

Supplement: Supplementary file 1 — Fig S1 [file JCMM-25-7720-s002.png]

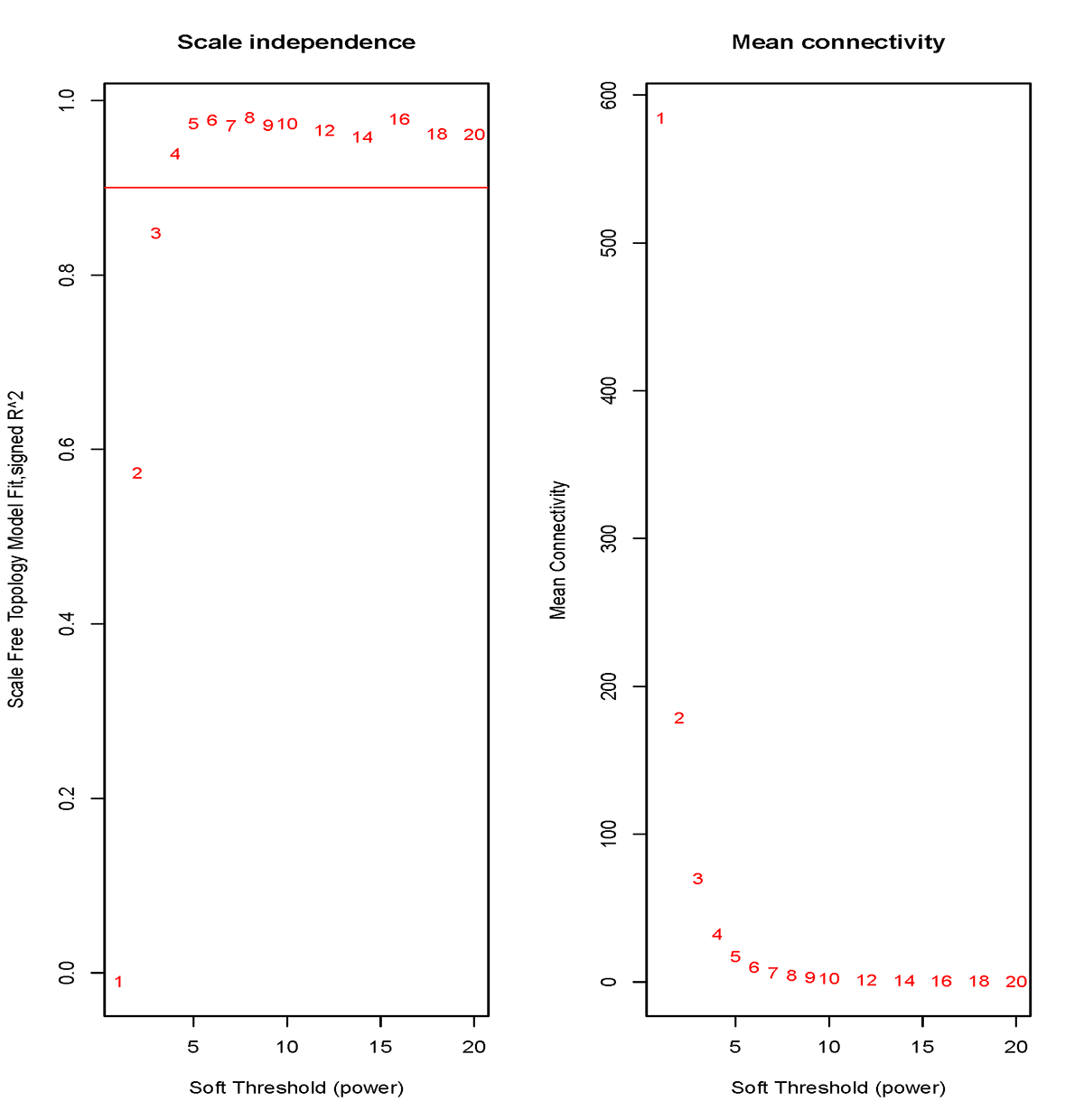

Supplement: Supplementary file 2 — Fig S2 [file JCMM-25-7720-s004.png]

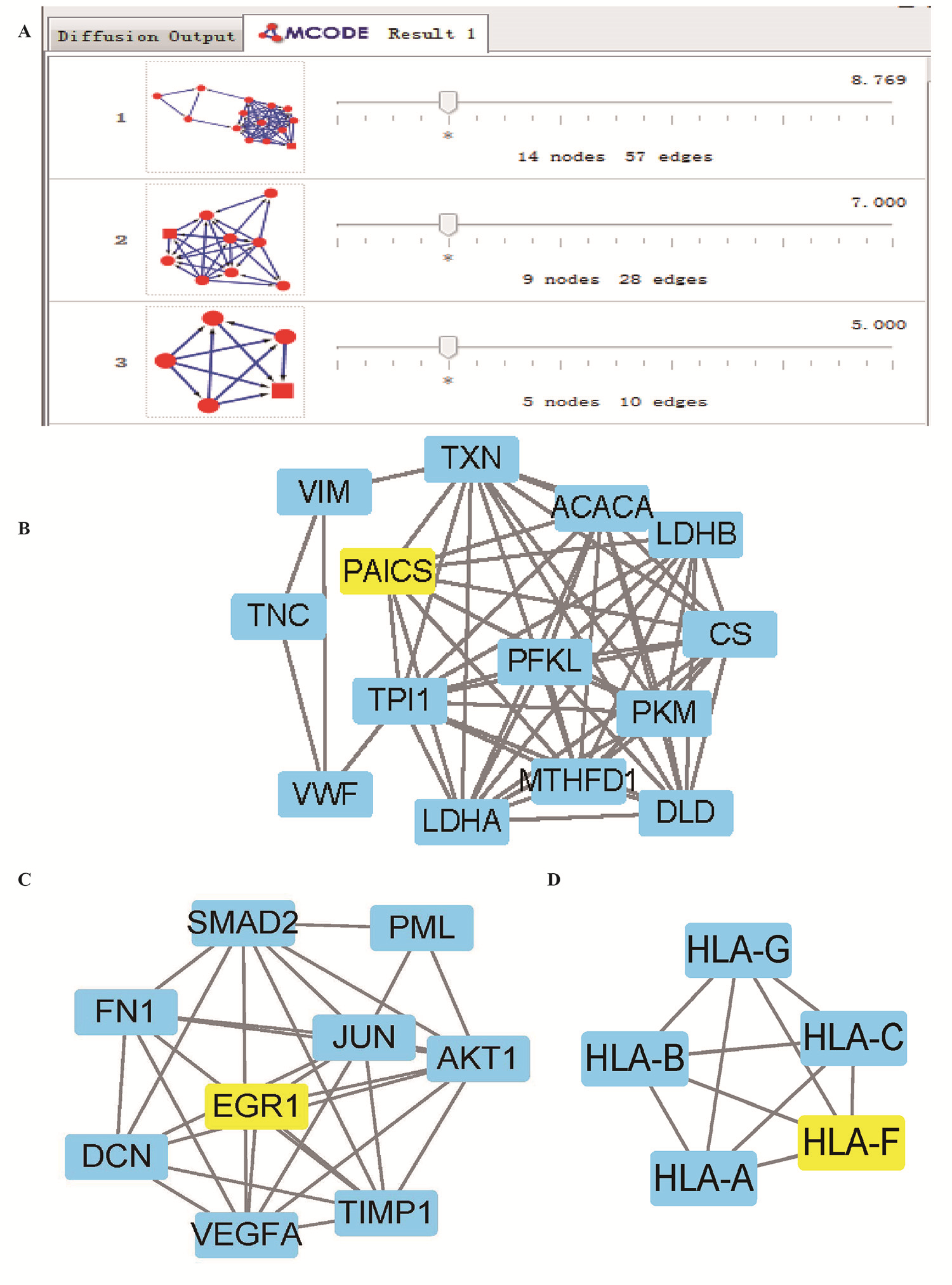

Supplement: Supplementary file 3 — Fig S3 [file JCMM-25-7720-s005.png]

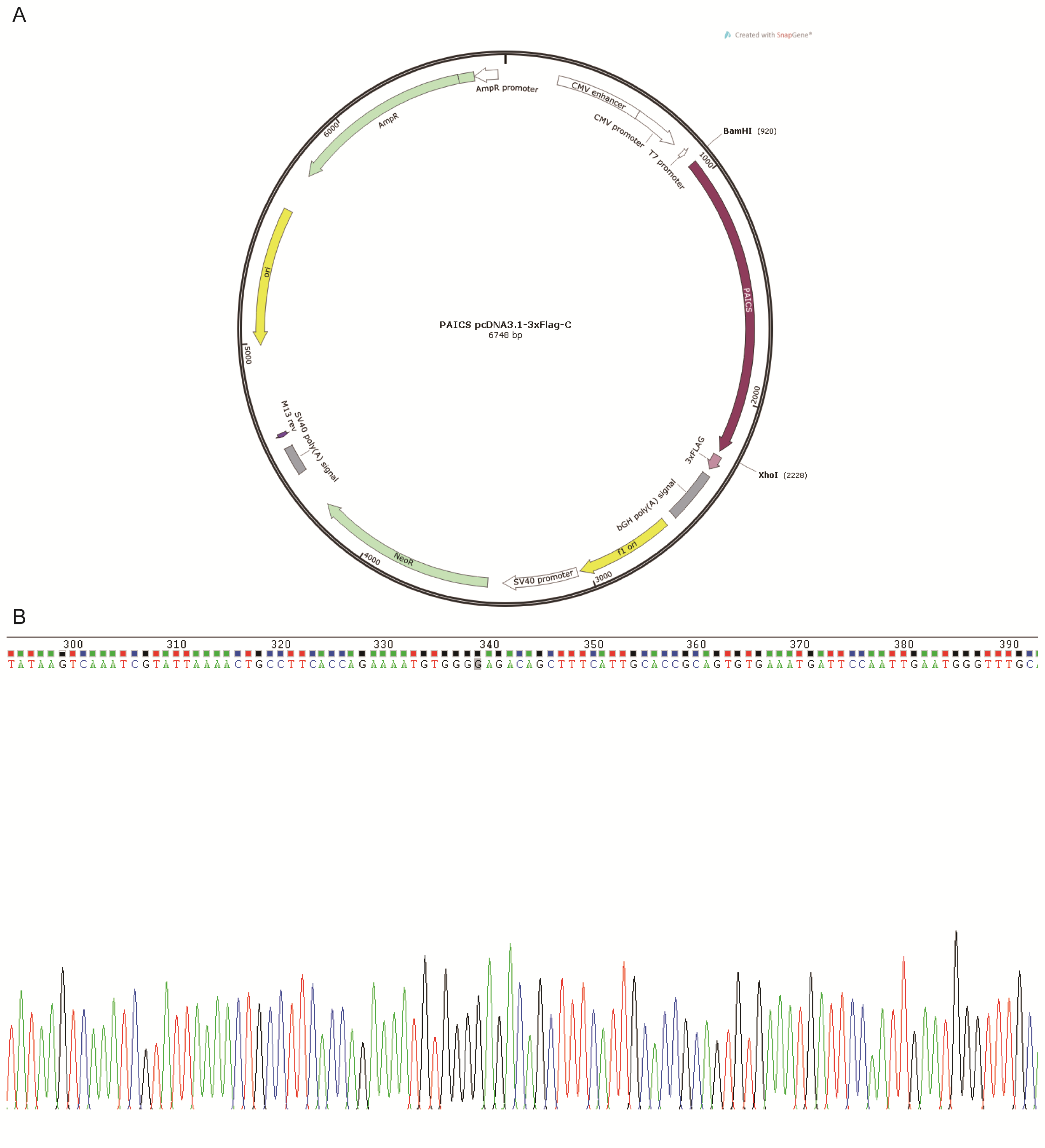

Supplement: Supplementary file 4 — Fig S4 [file JCMM-25-7720-s003.png]
